# 163. Assessment of Pneumonia FilmArray Use on the Impact on Antimicrobial De-escalation Within an Integrated Health System

**DOI:** 10.1093/ofid/ofac492.241

**Published:** 2022-12-15

**Authors:** Brittani Weichman, Amanda Bushman, David A Terrero Salcedo

**Affiliations:** UnityPoint Health Meriter, Madison, Wisconsin; UnityPoint Health Des Moines, Urbandale, Iowa; UNITYPOINT CLINIC, DES MOINES, Iowa

## Abstract

**Background:**

BioFire® FilmArray® Pneumonia Panel (PFA) is a highly sensitive and specific diagnostic tool. PFA has been found to reduce antimicrobial utilization, however, inappropriate use can lead to high positivity rates that often do not correlate with standard cultures. Injudicious PFA utilization has been associated with inappropriate antibiotic use. We aimed to evaluate the inpatient utilization of PFA, establish culture correlation and assess clinical decision by ordering providers based on these results.

**Methods:**

Retrospective review and descriptive analysis of adult patients presenting to an integrated health system in the Des Moines Metro, between March 2021 and September 2021, who had a PFA ordered and collected in their hospitalization. Indication, collection date, identified targets of PFA and cultures, antibiotic use, and trends in antibiotic de-escalation were collected.

**Results:**

A total of 127 PFA were collected, of which 94 were from sputum (74%) and 33 were from BAL (26%). From PFA done in BAL, 42% were collected outpatient. Timing of PFA collection in relation to admission date is shown in Table 1.

Sensitivity of PFA when matched to culture was 90% in sputum vs 67% in BAL, with specificity in sputum of 48% vs 82% in BAL.

The overall relative frequency of timing of PFA orders in relation to admission is shown in Figure 1. Of 127 PFA collected, 39 (31%) were on standard therapy for community acquired pneumonia at time of collection and 24/39 (19%) were collected within the first 48 hours of admission. We found that 27 (21%) patients had appropriate changes in antibiotics after PFA results and 42 (33%) were not changed when it was considered appropriate to do so. Out of 72 patients with negative PFA, 30 (42%) had no change in antibiotics.

**Figure:**
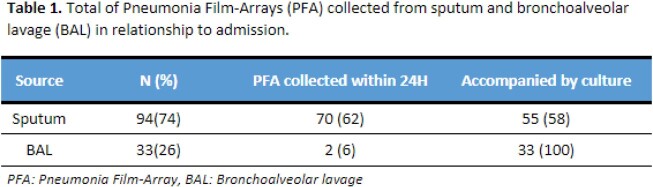

**Figure:**
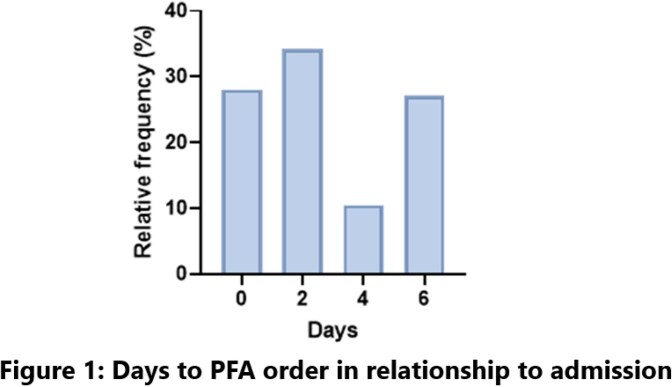

**Conclusion:**

PFA collected early in the hospitalization did not result in antibiotic de-escalation when appropriate and many times was ordered when results would not lead to de-escalation due to narrow standard of care therapy already in use. Within our health system, PFA is not adequately utilized to support decisions for antimicrobial de-escalation.

**Disclosures:**

**All Authors**: No reported disclosures.

